# Advances in the field of genetics and difficulties in the diagnosis of di george syndrome.

**DOI:** 10.1192/j.eurpsy.2023.2091

**Published:** 2023-07-19

**Authors:** I. E. Menendez Gil, M. L. Maria Del Carmen

**Affiliations:** 1 Psychiatry, virgen del rocio hospital, Sevilla; 2 Psychiatry, gregorio marañon hospital, Madrid, Spain

## Abstract

**Introduction:**

The spectacular progress of the last decade in the field of genetics is allowing a new development of medicine and the ability to make a better diagnosis. A great example of this is the diagnosis of chromosome 22q11 deletion, which occurs in 1:4000 live births.

**Objectives:**

This case wants to illustrate the difficulties in the diagnosis, despite technological advances.

**Methods:**

Exhaustive review of the literature

**Results:**

This is a 38-year-old male patient diagnosed with chromosome 22q11 deletion in adulthood.

Family history of medical problems: mother with genetic diagnosis of chromosome 22q11 deletion, in adulthood, after the diagnosis of her own son.

Personal history of medical problems:

- Psychiatry: he has been followed up intermittently in psychology since he was 6 years old, due to cognitive difficulties and behavioral alterations. He has had several hospital admissions in psychiatry during adolescence for behavioral disorders and intellectual disability, with possible psychotic symptoms. In treatment with antiepileptics and antipsychotics.

- Cardiology: aortic aneurysm and bicuspid aortic valve were detected. The patient underwent surgery in 2018.

- Genetics: he is diagnosed with chromosome 22q11 deletion in 2019. This is an inherited mutation of maternal origin that is detected later.

- Rheumatology: seropositive rheumatoid arthritis, non-erosive.

- Rehabilitation: treatment to improve psychomotor skills, from 6-12 years of age.

It is important to emphasize that the diagnosis was made at the age of 35 years, after a more deep study which had been carried out after the debut of the cardiac pathology. In addition, it is very striking that the diagnosis of his mother was made later than the one of the patient himself.

Currently, the patient presents serious difficulties in respecting the rules of coexistence at home and in understanding social norms, so that he has not been able to integrate in any environment and remains isolated at home. Serious behavioral alterations with tendency to physical and verbal heteroaggressiveness, difficulty in accepting limits and sexualized and uninhibited behaviors.

Clinical judgment: chromosome 22q11 deletion.

**Conclusions:**

Early diagnosis is essential to be able to treat and, above all, prevent the possible complications that this syndrome may present. However, diagnosis is sometimes very complex, despite advances in molecular diagnostic techniques. Therefore, an integrative approach is very valuable, looking at the individual as a whole and not only by systems or medical subspecialties. In addition, it would be very interesting to establish a means of communication between specialties. Finally, it would be a real step forward to integrate all the medical information of each person in a single medical record, an apparently simple aspect, but so far from being possible.

**Disclosure of Interest:**

None Declared

